# Diagnostic value of protein S100b as predictor of traumatic intracranial haemorrhage in elderly adults with low-energy falls: results from a retrospective observational study

**DOI:** 10.1007/s00068-023-02324-7

**Published:** 2023-07-13

**Authors:** Rebecca Wania, Alina Lampart, Sandra Niedermeier, Robert Stahl, Christoph Trumm, Paul Reidler, Christian Kammerlander, Wolfgang Böcker, Matthias Klein, Vera Pedersen

**Affiliations:** 1 Department of Orthopedics and Trauma Surgery, Musculoskeletal University Center Munich (MUM), University Hospital, LMU Munich, Marchioninistr 15., 81377 Munich, Germany; 2 Department of Medicine, Kantonsspital Lucerne, Spitalstrasse, 6000 Lucerne, Switzerland; 3 Department of Anaesthesiology and Intensive Care Medicine, ISAR Klinikum, Sonnenstr. 24–26, 80331 Munich, Germany; 4 Institute of Diagnostic and Interventional Neuroradiology, University Hospital, LMU Munich, Marchioninistr. 15, 81377 Munich, Germany; 5 Department of Radiology, University Hospital, LMU Munich, Marchioninistr. 15, 81377 Munich, Germany; 6 Trauma Hospital Styria, Goestinger Straße 24, 8020 Graz, Austria; 7 Department of Neurology, University Hospital, LMU Munich, Marchioninistr. 15, 81377 Munich, Germany; 8 Emergency Department, University Hospital, LMU Munich, Marchioninistr. 15, 81377 Munich, Germany

**Keywords:** Traumatic intracranial haemorrhage, Protein S100b, Older adult, Low-energy fall, Computed tomography, Anticoagulation therapy

## Abstract

**Purpose:**

The objectives of this study were to analyse the clinical value of protein S100b (S100b) in association with clinical findings and anticoagulation therapy in predicting traumatic intracranial haemorrhage (tICH) and unfavourable outcomes in elderly individuals with low-energy falls (LEF).

**Methods:**

We conducted a retrospective study in the emergency department (ED) of the LMU University Hospital, Munich by consecutively including all patients aged ≥ 65 years presenting to the ED following a LEF between September 2014 and December 2016 and receiving an emergency cranial computed tomography (cCT) examination. Primary endpoint was the prevalence of tICH. Multivariate logistic regression models and receiver operating characteristics were used to measure the association between clinical findings, anticoagulation therapy and S100b and tICH.

**Results:**

We included 2687 patients, median age was 81 years (60.4% women). Prevalence of tICH was 6.7% (180/2687) and in-hospital mortality was 6.1% (11/180). Skull fractures were highly associated with tICH (odds ratio OR 46.3; 95% confidence interval CI 19.3–123.8, *p* < 0.001). Neither anticoagulation therapy nor S100b values were significantly associated with tICH (OR 1.14; 95% CI 0.71–1.86; OR 1.08; 95% CI 0.90–1.25, respectively). Sensitivity of S100b (cut-off: 0.1 ng/ml) was 91.6% (CI 95% 85.1–95.9), specificity was 17.8% (CI 95% 16–19.6), and the area under the curve value was 0.59 (95% CI 0.54 − 0.64) for predicting tICH.

**Conclusion:**

In conclusion, under real ED conditions, neither clinical findings nor protein S100b concentrations or presence of anticoagulation therapy was sufficient to decide with certainty whether a cCT scan can be bypassed in elderly patients with LEF. Further prospective validation is required.

**Supplementary Information:**

The online version contains supplementary material available at 10.1007/s00068-023-02324-7.

## Background

Low-energy falls (LEF) (e.g. from standing height, chair, bed or less than 5 steps from stair) are amongst the most frequent causes for presentation to a primary care physician or the emergency department (ED) in elderly adults. Head injury, including traumatic intracranial haemorrhage (tICH), is considered to be the most common fall-related injury in this population [1]. Both morbidity and mortality after falls appear to increase with age [2]. Trauma registry analysis reveals that severe head injuries are common in elderly trauma victims, mainly due to falls from low levels [3]. In agreement with other authors, we found a prevalence for tICH in elderly adults with LEF presenting to the ED between 5 and 7% [4–6], whilst observed in-hospital mortality in patients with tICH due to LEF ranged from 8% [4] to 18% [6]. Although anticoagulation and antiplatelet therapies are considered to increase the risk for tICH [7–9], more recent studies do not support such an association [4, 6, 10, 11].

Consensus guidelines and clinical decision rules either lack specific recommendations for performing cranial computed tomography (cCT) in elderly adults with LEF and signs of head trauma [12–14] or recommend a cCT in every head trauma patient aged 65 years or older [15]. In addition, biomarkers like the calcium-binding protein S100b, which is largely but not exclusively expressed in brain astrocytes and released upon head injury [16, 17], have been shown to be correlated with CT abnormalities in trauma patients with mild traumatic brain injury, when sampled within 3–12 h after the injury [16–20]. The serum biomarker protein S100b has been added to the Scandinavian Guidelines for Initial Management of Minimal, Mild and Moderate Head Injuries for determination of need for cCT imaging in the low-risk group of patients [18, 21]. It accurately predicts normal cCT findings after minor head injury in elderly adults [22]. The determination of serum levels of protein S100b with a cut-off threshold of 0.1 ng/ml is an integral part in guidelines [18] and local protocols for management [16] of adult minor head trauma. However, it remains unclear from previous data whether protein S100b is a reliable negative prediction tool for tICH in elderly adults with LEF in real-life ED circumstances, when in the majority of patients’ protein S100b, sampling cannot be provided in the 3 to 6 h interval from the index fall.

Therefore, the objective of this study was to analyse the clinical value of protein S100b in association with clinical findings and anticoagulation/antiplatelet medication in predicting tICH and unfavourable outcomes in elderly individuals with LEF.

## Methods

### Study design and setting

This retrospective single-centre observational study represents a subset of a published larger patient cohort [23], including patients from one university tertiary care hospital in Germany (University Hospital of Ludwig-Maximilians-University Munich) using electronic health records (EHR). The study is in accordance with the declaration of Helsinki and was conducted using STROBE guidelines. Ethics approval was obtained from the local ethics committee (EK LMU 17-217, approved May 10, 2017).

### Study population

Parts of the methods used in this study have been previously described [24]. In short, patients aged 65 years and older, who suffered from a LEF (as previously defined [24]: standing height, low level furniture, low level ≤ 1 m) and underwent cCT examination within 48 h of the index visit to the ED from September 1st 2014, to December 31st 2016, were consecutively included. Patients referred from another hospital with preceding imaging, patients who required trauma team activation, as well as patients with a delayed presentation (≥ 8 days after the fall) were excluded from this study. A subgroup of patients presenting from January 1, 2016, to December 31, 2016 has been previously analysed and published [4].

### Data collection

The radiology information system (RIS) of the study centre was screened for patients aged 65 years and older, receiving a CT examination within 48 h of their index visit to the ED during the above mentioned 28-months-period [24]. EHR of retrieved cases were manually screened for documented LEF by two trained non-blinded reviewers (R.W., A.L. S.N. and V.P.). Baseline demographics, in-hospital mortality, Emergency Severity Index (ESI), Glasgow Coma Scale (GCS) on initial presentation, loss of consciousness (LOC), visible signs of injuries above the clavicles (e.g. hematoma, bruises, wounds), symptoms of head injury (amnesia, headache, vertigo, nausea, vomiting), final injury diagnosis, polypharmacy (more than five medications), laboratory values of the initial routine blood sampling upon presentation to the ED (serum calcium-binding protein S100b, serum albumin, serum sodium, serum urea, serum creatinine, thrombocytes in cell blood count, activated partial thromboplastin time, aPTT and international normalised ratio INR in citrate blood measurement) and anticoagulation/antiplatelet therapy were extracted from the EHR. Anticoagulation therapy was distinguished as previously described [4]: oral anticoagulants (OACs; phenprocoumon or acenocoumarol), direct oral anticoagulants (DOACs; dabigatran, rivaroxaban, apixaban or edoxaban), heparins (liquemin or low-molecular-weight heparins), antiplatelet agents (acetylsalicylic acid, clopidogrel, ticagrelor or prasugrel), single or dual therapy and no anticoagulation/antiplatelet therapy. A tICH was classified as epidural hematoma, subdural hematoma, traumatic subarachnoid haemorrhage or intraparenchymal haemorrhage. Fractures of the skull and maxillofacial or cervical spine fractures were extracted from the final radiology report. Chart abstraction for all parameters was performed for all patients by two trained observers (R.W., S.N., A.L. and V.P.) in every case. Disagreement or equivocalness was decided upon by a third observer (R.W. and V.P.).

The Injury Severity Score (ISS; range: 1–75) was calculated based on the Abbreviated Injury Scale (AIS) [25], extracted from the final radiology and discharge report or directly from cCT images, performed by a senior trauma surgeon (V.P.). Using the highest AIS (range: 1–6) of the three (AIS_A_, AIS_B_, AIS_C_) most severely injured body regions, the ISS was determined as: ISS = AIS_A_^2^ + AIS_B_^2^ + AIS_C_^2^. Head-specific AIS (hAIS) was defined by the most severe cranial/intracranial injury.

Screening and chart review abstraction were conducted in accordance with the recommendations for medical chart review [26, 27], which were fulfilled for 11 of 12 guidelines (abstractors were not blinded to the hypothesis). Double-data entry was performed in a Microsoft Access 2010/2016 database (Microsoft, Redmond, Washington, USA).

### Key outcome measures

The primary endpoint of the study was the endpoint of a tICH. Secondary endpoints were head-specific AIS in case of an intracranial haemorrhage, in-hospital mortality and neurosurgical intervention in case of tICH.

### Predictor variables

Definition of predictor variables was carried out as previously described [4]. In short, any anticoagulation or antiplatelet therapy, age, sex, ESI level, GCS, presence of polypharmacy, reported LOC, symptoms of head injury, injury signs above the clavicles, newly diagnosed fractures of the skull, maxillofacial or cervical spine and laboratory values (S100b, serum albumin, serum sodium, serum urea, serum creatinine and INR) were predictor variables for tICH. In a further step, anticoagulation/antiplatelet therapy at three different levels of granularity was the predefined predictor variables. First, any anticoagulation or antiplatelet therapy vs. no anticoagulation or antiplatelet therapy. Second, antiplatelet therapy exclusively, anticoagulation therapy exclusively and combination of anticoagulation and antiplatelet vs. no anticoagulation/antiplatelet therapy. Third, single antiplatelet therapy exclusively, dual antiplatelet therapy exclusively, OAC exclusively, DOACs exclusively, combination of OAC and antiplatelet, and combination of direct-acting oral anticoagulants and antiplatelet vs no anticoagulation/antiplatelet therapy. Group sizes for heparins (*n* = 19) and for combinations of more than one anticoagulation (*n* = 3) or combinations of heparins with any antiplatelet agent (*n* = 17) or combination of more than one anticoagulation with an antiplatelet agent (*n* = 0) were small; therefore, these groups were excluded from further analysis (see Table 1).

**Table 1:** Baseline characteristics of 2687 older adult patients presenting with low-energy falls and cCT from 1 September 1, 2014 to December 31, 2016

All patients (n = 2687)	
Age (median, IQR)	81 (75–88)
Females (%)	1624 (60.4)
Hospital admission (%)	1740 (64.8)
In-hospital mortality (%)	55 (2.0)
ICU admission (%)	107 (4.0)
Delay to ED presentation (%)	
≤ 24 h	2469 (91.9)
24–48 h	109 (4.1)
48 h to 7 days	109 (4.1)
tICH (%)	180 (6.7)
Subdural hematoma (%)	116 (4.3)
Subarachnoidal haemorrhage (%)	76 (2.8)
Epidural hematoma (%)	7 (0.3)
Intraparenchymal haemorrhage (%)	51 (1.9)
hAIS ≥ 3 (% of tICH)	127 (70.6)
ICU (% of tICH)	25 (14.0)
Neurosurgical intervention (% of tICH)	22 (12.2)
Mortality (% of tICH)	11 (6.1)
Any AC/AP medication (%)	1386 (51.6)
AP medication (%)	830 (30.9)
Dual AP medication (%)	57 (2.1)
AC (%)	457 (17.0)
OAC (%)	193 (7.2)
DOAC (%)	242 (9.0)
Heparins (%)	19 (0.7)
> 1 AC (%)	3 (0.1)
AC & AP medication (%)	99 (3.7)
OAC & AP medication (%)	36 (1.3)
DOAC & AP medication (%)	46 (1.7)
Heparins & AP medication (%)	17 (0.6)
> 1 AC & AP medication (%)	0 (0)
No AC/AP medication (%)	1084 (40.3)
Unknown AC/AP medication (%)	217 (8.1)
Polymedication (%)	991(36.9)
ISS (median, range, IQR)	2 (1–33, 1–5)
Non-injurious fall (%)	457 (17.0)
ESI (Median, IQR)	3 (3–3)
Supraclavicular injury sign (%)	1263 (47.0)
Skull bone fracture (%)	45 (1.7)
Facial bone fracture (%)	165 (6.1)
Cervical spine fracture (%)	42 (1.6)
Spine fracture (%)	72 (2.7)

*AC* anticoagulation, *AP* antiplatelet, *DOAC* direct oral anticoagulant, *ESI* emergency severity index, *hAIS* head-specific abbreviated injury scale, *ICU* intensive care unit, *IQR* interquartile range, *ISS* injury severity score, *OAC* oral anticoagulant, *tICH* traumatic intracranial haemorrhage

### Statistics

For descriptive statistics, the median and interquartile ranges (IQRs) were used to report continuous and ordinal data, where applicable. The Kruskal–Wallis test was used to compare central tendency for continuous and ordinal data. The Pearson’s chi-squared test with Yates’s continuity correction or the Fisher exact test was used for the comparison of categorical data. A first multivariable logistic regression, including all predictor variables, was calculated to identify risk factors for the primary outcome tICH. Of all identified statistically significant risk factors, ESI level below 5, GCS below 15, and injury signs above the clavicles were chosen as covariates for the subsequent regression models because they are easily assessable at triage. For the binary outcomes tICH and in-hospital mortality, multivariable logistic regression models were adjusted for ESI level, GCS and injury signs above the clavicles to assess the association with anticoagulation/antiplatelet therapy. For the outcome of in-hospital mortality, the multivariable model was additionally adjusted for age and sex. Multivariable quasi-Poisson regression models adjusted for ESI level, GCS and injury signs above the clavicles were calculated to assess the association between anticoagulation/antiplatelet therapy and continuous ordinal outcomes, respectively (head-specific injury severity, hAIS).

The receiver operating characteristic (ROC) analysis was performed to determine whether S100b can be used as a diagnostic tool for predicting tICH. The area under the ROC curve (AUC) was calculated with 95% confidence intervals (CI). Partial AUC (pAUC) was used to compare only a proportion of the AUC curve, which was set to the clinically relevant range of 90 to 100% sensitivity. The *P* values < 0.05 were considered significant. R software v.4.1.2 (https://www.R-project.org/) and RStudio 2022.07.1 Build 554 were used to carry out the statistical analysis.

## Results

**Fig. 1:**
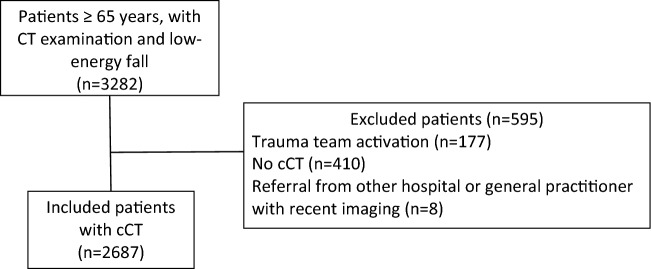
Inclusion and exclusion flow diagram of patient selection from September 1, 2014 to December 31, 2016, undergoing cranial computed tomography (cCT) examination of the head during emergency department presentation or within 48 h

We included 2687 patients in the analysis (Fig. 1). Median age was 81 years (IQR 75 − 88) and 60.4% of included patients were female. Table 1 shows baseline demographic information. Overall in-hospital mortality rate was 2.0% (55/2687), admission rate to intensive care unit (ICU) was 4.0% (107/2687) (Table 1).

Traumatic intracranial haemorrhage was detected in 180 of 2687 patients (6.7%). Of these 180 patients with tICH, 127 (70.6%) had a severe tICH (hAIS ≥ 3), 24 were admitted to the ICU (14.0%). Of 180 patients with tICH, 150 (83.3%) had an isolated head injury, 22 received a neurosurgical intervention (12.2%) and 11 (6.1%) died in the hospital (Table 1). Additional detailed information about patients with tICH receiving neurosurgical intervention or none is given in Supplementary Table 1.

Anticoagulation/antiplatelet therapy was documented in 1386 of 2687 (51.6%) patients, with a prevalence for tICH of 93 of 1386 (6.7%) vs. no anticoagulation/antiplatelet therapy in 1084 of 2687 (40.3%) patients, with a prevalence of 72 of 1084 (6.6%). In 217 of 2687 (8.1%) patients, the documentation about anticoagulation/antiplatelet therapy was missing, of which 15 of 217 (6.9%) patients were diagnosed with a tICH.

Analysis of the serum protein S100b concentrations upon presentation to the ED was performed in 1851 of 2687 patients of our study population (68.9%). 2469 (91.9%) of these patients patients presented within 24 h delay after the fall. In 1532 of 1851 patients (82.8%), serum S100b concentrations were elevated above 0.1 ng/ml, which determines the cut-off value for screening of tICH [16, 18]. However, exact time of sampling after the fall was not determined due to imprecise given information.

**Fig. 2:**
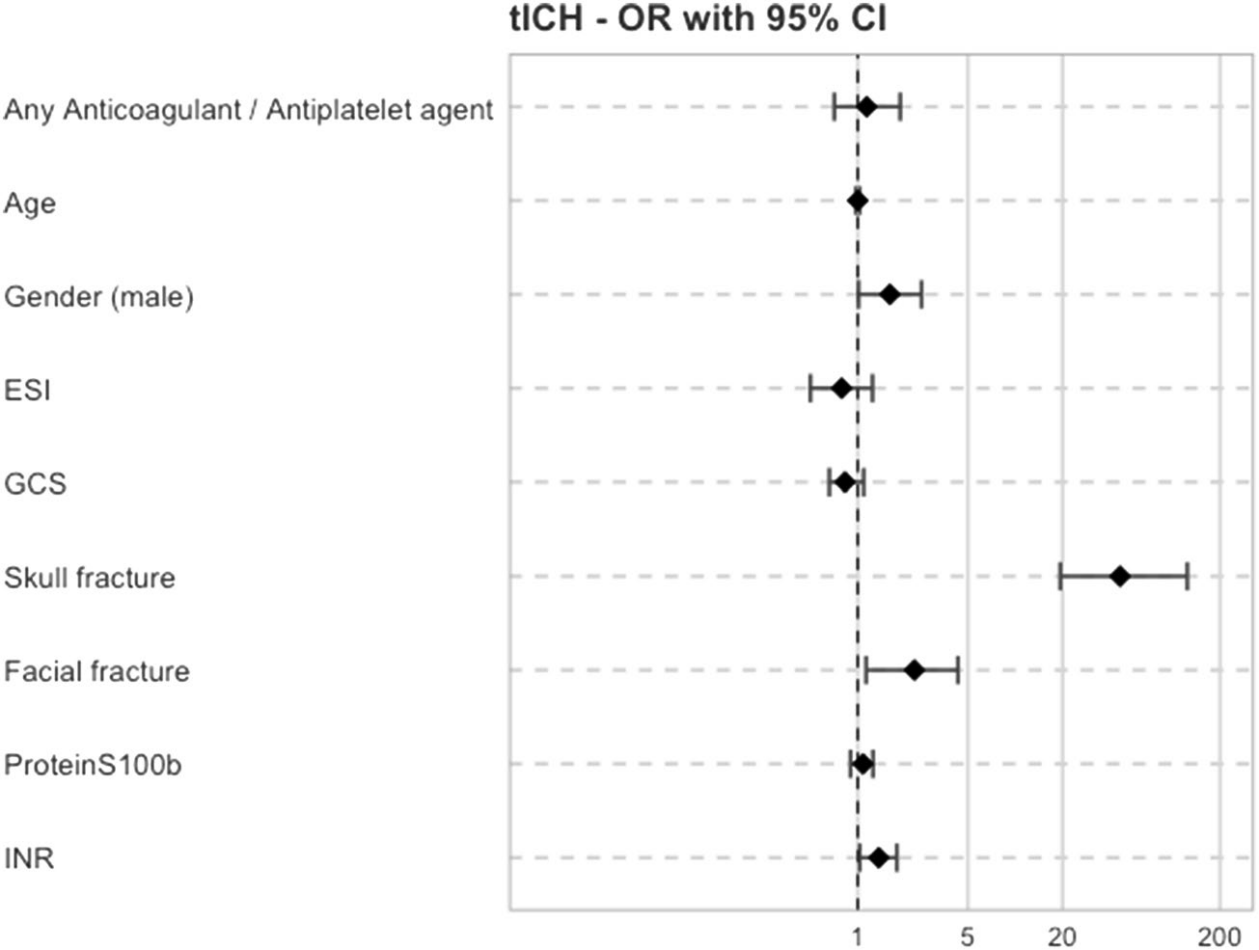
Multivariable logistic regression model for risk factors (odds ratio with 95% confidence interval) for the primary outcome traumatic intracranial haemorrhage (tICH). *ESI* emergency severity index, *GCS* glasgow coma scale, *INR* international normalised ratio

The multivariable logistic regression model for risk factors of tICH indicated skull fracture as the strongest predictor (OR 46.3; 95% CI 19.3–123.8, *p* < 0.001). Facial bone fracture (OR 2.29; 95% CI 1.33–4.33, *p* = 0.01), male gender (OR 1.60; 95% CI 1.01–2.54, *p* = 0.04) and increased INR value (OR 1.36; 95% CI 1.03–1.77, *p* = 0.02) were further predictive for tICH. GCS level (OR 0.83; 95% CI 0.66–1.09, *p* = 0.14) and ESI level (OR 0.79; 95% CI 0.50–1.24, *p* = 0.3) were not predictive for tICH (Fig. 2). Any anticoagulation or antiplatelet medication was not predictive for tICH (OR 1.14; 95% CI 0.71–1.86, *p* = 0.6). The biomarker S100b was not predictive (OR 1.08; 95% CI 0.90–1.25, *p* = 0.35) for tICH. Supplementary Table 2 summarises the underlying univariate logistic regression analysis of potential risk factors for tICH and severe tICH.

Multivariable regression models demonstrated that medication with any anticoagulation or antiplatelet agent was not associated with the primary outcome of tICH (OR 0.97; 95% CI 0.68–1.37; *p* = 0.85), in-hospital mortality (OR 1.96; 95% CI 0.95–4.34; *p* = 0.08), or ISS (incident rate ratio IRR: 0.97; 95% CI 0.88–1.07; *p* = 0.60) (Supplementary Fig. 1). In patients with tICH (180/2687), any anticoagulation or antiplatelet therapy was not associated with head-specific injury severity (IRR: 0.75; 95% CI 0.36–1.55. *p* = 0.43) (Supplementary Fig. 1). Anticoagulation combined with an antiplatelet agent was associated with in-hospital mortality after a fall (OR 4.82; 95% CI 1.40–14.8; *p* = 0.008). Combined treatment of an oral anticoagulant with an antiplatelet agent (OR 5.23; 95% CI 0.72–23.875; 0 = 0.052) or combined treatment of a direct-acting oral anticoagulant with an antiplatelet agent (OR 6.16; 95% CI 1.29–22.38; *p* = 0.009) was also associated with in-hospital mortality.

**Fig. 3:**
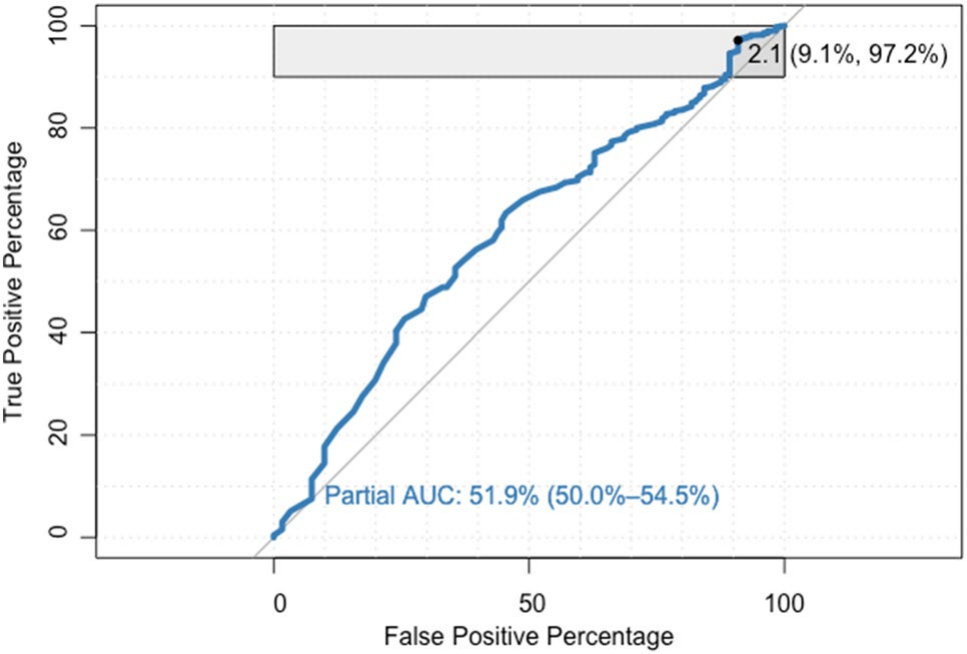
Receiver operating characteristic curve (ROC) demonstrating performance of serum calcium-binding protein S100b (S100b) as biomarker for predicting traumatic intracranial haemorrhage (tICH) on cCT scan within 48 h of the index fall. Illustrated is the partial area under the curve (pAUC) (with 95% confidence interval), with specificity and sensitivity set to 90–100% and the cut-off value of 2.1 ng/ml at the particular level of specificity/sensitivity

With a cut-off threshold of 0.1 ng/ml, sensitivity was 91.6% (CI 95% 85.1–95.9), specificity was 17.8% (CI 95% 16–19.6), positive predictive value (PPV) was 7.4% (CI 95% 7.9–7.8) and negative predictive value (NPV) was 96.7% (CI 95% 94.2–98.2) for predicting tICH. The AUC values of S100b were 0.59 (95% CI 0.54 − 0.64) for predicting tICH. The ROC curve showing the performance of S100b for prediction of tICH is presented in Fig. 3. With a cut-off threshold of 0.305 pg/ml, sensitivity was 63%, specificity was 54%, PPV was 95% and negative predictive NPV was 9.2% for predicting tICH. In 10 patients with a tICH detected by cCT and presenting within 24 h after the index fall, serum protein S100b concentrations were below the defined cut-off threshold of 0.1 ng/ml upon initial measurement. Characteristics of these patients are presented in Supplementary Table 3.

## Discussion

In this retrospective cohort analysis of a single-centre university tertiary care hospital, we found an overall prevalence of 6.7% for tICH in elderly patients with low-energy falls. Neither anticoagulation, antiplatelet therapy nor the combined treatment was relevant risk factors for tICH or severe intracranial haemorrhage. Conversely, concomitant skull fractures are highly associated with tICH. Although the calcium-binding protein S100b is valuable in determining the need for cCT in younger patients, the serum biomarker showed a poor performance in predicting tICH in this real-life cohort of elderly patients with exclusively low-energy falls. This study combines factors of clinical examination, the presence of an antiplatelet/anticoagulation therapy and the biomarker protein S100b for prediction of tICH in the elderly patient with LEF, where mild TBI is possible but not obvious in every case.

Comparable to our previously published analysis [4] and other recent results from a prospective observational study in a cohort of elderly adults with LEF [6], the prevalence for a tICH was 6.7%, confirming this substantial rate of partially relevant low-energy accident injury. In this present study cohort, an increased INR and concomitant skull fractures, detected by cCT scan, were associated with a tICH, whereas no other clinical finding or laboratory parameters proved to be significantly associated with tICH, in contrast to the prior published data [4, 6, 22]. However, the recent analysis adds more support to prior findings [4, 6, 10, 11, 22, 28, 29], that neither anticoagulation, antiplatelet therapy nor their combinations are relevant risk factors for tICH or severe tICH. Consequently, the absence of anticoagulation or antiplatelet therapy in patients’ medication does not allow for omission of cCT in elderly adults with LEF and suspected brain injury.

Elevated serum levels of the calcium-binding protein S100b, sampled within 3 to 12 h after the injury, have been shown to be correlated with CT abnormalities in trauma patients with mild traumatic brain injury [16–20]. The biomarker has been added to the Scandinavian Guidelines for Initial Management of Minimal, Mild and Moderate Head Injuries for determination of need for cCT imaging in the low-risk group of patients [18, 21]. Prior prospective studies, including all kinds of trauma mechanisms and all ages of trauma patients in the ED, analysed performance of protein S100b for the prediction of tICH [22, 30, 31] with cut-off levels of 0.1 ng/ml [31], 0.105 ng/ml [22, 32] or 0.2 ng/ml [30] and S100b sampling within 3 [22, 31, 32] to 4 [30] hours after the index accident. In older patients with mild traumatic brain injury, negative protein S100b levels sampled within 3 h after the impact predicted a normal cCT with an AUC of 0.73 (CI 0.67–0.79), a sensitivity of 98.0% (CI 89.5–99.7%), a specificity of 35.3% (CI 31.9–38.8%), a NPV of 99.6% (CI 97.9–99.9%) and a PPV of 9.4% (CI 7.2–12.2%) [22]. Accordingly, classification performance for detecting tICH in cCT yielded a sensitivity of 100% (95% CI 63–100), a specificity of 5% (95% CI 2–13), a NPV of 100% (CI 40–100%), and a PPV of 11% (CI 5–20%) with an AUC of 0.78 (CI 0.67–0.89) [30]. Elevated S100b levels were more common in the group of elderly adults [31, 32], aged 65 years or older, whilst specificity for CT findings was higher in the subgroup of younger adults (35%) vs. 30% in the entire population, with a sensitivity of 100% [31]. Mean S100b serum levels differ in different types of tICH when measured within 3 h after the injury, i.e. S100b is the highest in patients with cerebral oedema and lowest in patients with concussion symptoms and no traumatic findings in cCT [32]. The analysis of our study revealed no significant association of S100b levels with tICH detected in cCT, with poorer performing characteristics of the biomarker in the prediction of tICH (AUC: 0.59) and lower sensitivity and specificity than in prior studies [22, 30]. Noteworthy, 10 out of 180 patients with tICH (5 of them with severe tICH with a hAIS ≥ 3) had no elevated serum levels of protein S100b, although sampled within 24 h after the index fall. Half-life of protein S100b is known to be 2 to 6 h in mild traumatic brain injury and 24 h in severe brain injury [33, 34], with an optimal time interval for screening of 1–3 h after the head impact [35]. We interpret our findings mainly in the light of the imprecise information given about the exact time point of the index fall, which was rarely within the 1–3 h interval. Therefore, this might limit the clinical value of protein S100b as a screening biomarker in real-life cohorts of elderly adults with LEF and unknown or imprecise delay between injury and presentation to the ED.

Considering protein S100b as a screening tool in the ED setting to reduce the number of CT scans and associated costs, two prior studies analysed the economic impact of protein S100b as pre-cCT screening tests [36, 37]. Inclusion of protein S100b in the Scandinavian guidelines [18, 21] with strict adherence during decision making, resulted in a cost-saving of up to 71 € per patient with suspected mild traumatic brain injury [36]. Waiting time for blood test or final CT scans were not considered in this study. Protein S100b becomes cost-lowering when the proportion of patients undergoing a cCT scan is high (> 78%) or the waiting time for CT scan results exceeds the waiting time for blood test results by 96 min [37]. Despite its ability to reduce costs, the performance of protein S100b to predict tICH is limited. Our data support the consideration, that protein S100b determination can be dispensed in the ED work-up of elderly adults with LEF and risk for tICH. However, as there are no reliable alternative clinical risk factors identified recently, no recommendations can be made to reduce cCT examinations in these patients.

The main strength of our study is the observational design with a representative cohort of a local population of elderly adults and meaningful sample sizes. This allowed the calculation of multivariable regression models for the primary outcome and subgroups of anticoagulation and antiplatelet therapies. Nevertheless, the study is limited by its retrospective design and by initial patient selection. The first limitation is that the main clinical findings were extracted from EHR, which makes the study dependent on documentation quality and granularity. Second, data are derived from a single centre. Thus, no conclusions can be drawn on the overall population of elderly adults with LEF. Third, the number of neurosurgical interventions, defining a clinically important brain injury, was too small to allow for a statistical meaningful analysis of risk and prediction factors. Despite in-hospital mortality of patients with tICH having increased more than threefold, the majority of these patients died from reasons other than acute tICH. This further limits the significance of the prediction model with regard to unfavourable outcomes due to tICH.

In conclusion, we found a prevalence of 6.7% for tICH in elderly adults presenting to the ED after a LEF. Determination of serum concentrations of the calcium-binding protein S100b does not allow reliable prediction of tICH if the exact time point of the trauma is unclear. Our data suggest that screening for clinical findings or biomarkers is not sufficient to safely decide for omission of cCT scan in elderly patients after LEF, neither is the prevalence of anticoagulation nor antiplatelet therapy. These findings merit further prospective validation.

### Supplementary Information

Below is the link to the electronic supplementary material.

Supplementary file1 (DOCX 105 KB)

## Data Availability

The datasets used and/or analysed during the current study are available from the corresponding author on reasonable request.
