# Supplementary material for: Diagnostic value of protein S100b as predictor of traumatic intracranial haemorrhage in elderly adults with low-energy falls: results from a retrospective observational study
Source: Eur J Trauma Emerg Surg. 2023 Jul 13;50(1):205–13. doi: 10.1007/s00068-023-02324-7 (PMC10924004; doi:10.1007/s00068-023-02324-7)
Supplement: Supplementary file 1 — Supplementary file1 (DOCX 105 KB) [file 68_2023_2324_MOESM1_ESM.docx]

**Supplementary Table 1.** Demographic and clinical characteristics of patients with traumatic intracranial hemorrhage (tICH) with and without neurosurgical intervention, compared to patients without tICH.

| **Variable** | **tICH (n = 180)** | **tCH with neurosurgery (n = 22)** | **tICH without neurosurgery (n = 158)** | **No tICH (n = 2507)** |
| --- | --- | --- | --- | --- |
| Age (Median, IQR) | 83 (75-88) | 75.5 (74-84) | 83 (76-88) | 81 (75-88) |
| Age 65-75 (%)  Age 75-85 (%)  Age > 85 (%) | 39 (21.8)  72 (40.2)  69 (38.3) | 9 (41)  10 (45)  3 (13.6) | 30 (19)  62 (39.2)  66 (41.8) | 553 (22.0)  1034 (41.2)  920 (36.7) |
| Sex (male) (%) | 89 (49.7) | 14 (63.6) | 75 (47.5) | 974 (38.8) |
| Delay ≤ 24 h (%)  Delay 24-48h (%)  Delay 48h-7d (%) | 157 (87.2)  8 (4.5)  15 (8.4) | 17 (77.3)  0 (0)  5 (22.7) | 140 (88.6)  8 (5.1)  10 (6.3) | 2312 (92.2)  101 (4.0)  94 (3.8) |
| Hospital admission (%)  ICU admission (%) | 169 (93.9)  25 (14.0) | 22 (100)  10 (45.5) | 147 (93)  15 (9.5) | 1571 (62.7)  82 (3.3) |
| In-hospital mortality (%) | 11 (6.1) | 1 (4.5) | 10 (6.3) | 44 (1.8) |
| GCS (Median, IQR)  GCS<14 (%) | 15 (14-15)  18 (10) | 15 (14-15)  2 (9.1) | 15 (14-15)  16 (10.2) | 15 (14-15)  96 (3.8) |
| ISS (Median, IQR) | 10 (8.8-17) | 25 (21.25-25) | 9 (6.5-16) | 2 (1-4) |
| Unconsciousness | 18 (10) | 2 (9.1) | 16 (10.1) | 172 (6.9) |
| Supraclavicular injury signs (%) | 94 (52.2) | 3 (13.6) | 91 (57.6) | 1169 (46.6) |
| Commotio signs | 50 (27.8) | 9 (40.9) | 41 (25.9) | 450 (17.9) |
| ESI (Median, IQR) | 3 (3-3) | 3 (2.8-3) | 3 (3-3) | 3 (3-3) |
| Cervical spine fracture (%) | 4 (2.2) | 0 (0) | 4 (2.5) | 38 (1.5) |
| Spine fracture | 6 (3.4) | 0 (0) | 6 (3.8) | 66 (2.6) |
| Skull bone fracture (%) | 33 (18.4) | 5 (22.7) | 28 (17.7) | 12 (0.5) |
| Facial bone fracture (%) | 22 (12.3) | 1 (4.5) | 21 (13.3) | 143 (5.7) |
| Polymedication (%) | 66 (36.7) | 9 (40.9) | 57 (36.1) | 925 (36.9) |
| Any AC/AP (%) | 93 (51.7) | 17 (77.3) | 76 (48.1) | 1293 (51.6) |

AC/AP: anticoagulation/antiplatelet medication; GCS: Glasgow Coma Scale; ICU: intensive care unit; IQR: interquartile range; ISS: injury severity scale

**Supplementary Table 2.** Univariate logistic regression analysis of potential risk factors for tICH in the whole study population (n=2687) and for severe head injury (hAIS ≥ 3) in patients with tICH (n=180).

|  | **tICH (n=2687)** |  |  | **hAIS (n=180)** |  |  |
| --- | --- | --- | --- | --- | --- | --- |
| **Variable** | **Unadjusted OR** | **95% CI** | **p-value** | **Unadjusted OR** | **95% CI** | **p-value** |
| Age | 0.99 | 0.97-1.01 | 0.35 | 1.01 | 0.97-1.05 | 0.8 |
| Age 65-75  Age 75-85  Age > 85 | Reference  1.01  0.96 | 0.67-1.51  0.63-1.42 | 0.95  0.83 | Reference  1.12  1.06 | 0.48-2.71  0.45-2.59 | 0.79  0.89 |
| Sex (male) | 1.56 | 1.15-2.11 | 0.0043* | 1.19 | 0.63-2.28 | 0.59 |
| Delay  ≤24h  24-48 h  48h-7 d | Reference  1.17  2.37 | 0.52-2.31  1.29-4.06 | 0.67  0.003 | Reference  0.30  0.33 | 0.02-1.77  0.05-1.24 | 0.27  0.15 |
| GCS  GCS<14 | 0.81  2.70 | 0.73-0.92  1.54-4.50 | <0.001  <0.001 | 0.95  0.80 | 0.71-1.29  0.25-2.28 | 0.72  0.7 |
| Unconsciousness | 1.52 | 0.8-2.47 | 0.11 | 1.59 | 0.56-4.30 | 0.36 |
| Supraclavicular injury signs | 0.79 | 0.58-1.07 | 0.13 | 0.57 | 0.29-1.09 | 0.09 |
| Commotio signs | 1.29 | 0.62-3.02 | 0.52 | 3.39 | 0.54-66.27 | 0.27 |
| ESI | 0.66 | 0.52-0.85 | <0.001 | 1.27 | 0.77-2.16 | 0.35 |
| ISS | 1.37 | 1.33-1.43 | <0.001 | 1.37 | 1.33-1.43 | <0.01 |
| Cervical spine fracture | 0.67 | 0.27-2.26 | 0.45 | 1.27 | 0.16-25.96 | 0.84 |
| Spine fracture | 0.78 | 0.36-2.04 | 0.57 | 2.15 | 0.34-41.71 | 0.49 |
| Skull bone fracture | 47.01 | 24.42-96.60 | <0.001 | 3.66 | 1.35-12.86 | <0.05 |
| Facial bone fracture | 2.31 | 1.40-3.66 | <0.001 | 0.37 | 0.15-0.91 | <0.05 |
| Polymedication | 1.03 | 0.75-1.41 | 0.87 | 1.09 | 0.56-2.11 | 0.8 |
| ProteinS100b | 1.19 | 1.05-1.36 | <0.05 | 0.54 | 0.2-1.03 | 0.12 |
| INR | 1.28 | 1.04-1.52 | <0.05 | 0.89 | 0.54-1.28 | 0.57 |
| Serum Creatinine | 0.95 | 0.75-1.06 | 0.59 | 0.94 | 0.55-1.41 | 0.76 |
| Any AC/AP | 1.00 | 0.73-1.38 | 0.99 | 0.66 | 0.34-1.30 | 0.23 |
| AC | 0.94 | 0.62-1.47 | 0.95 | 0.35 | 0.11-0.95 | 0.053 |
| AP | 1.06 | 0.74-1.55 | 0.74 | 0.84 | 0.38-1.78 | 0.64 |
| OAC | 0.79 | 0.46-1.43 | 0.41 | 0.43 | 0.09-1.50 | 0.22 |
| DOAC | 1.16 | 0.66-2.17 | 0.63 | 0.31 | 0.05-1.27 | 0.14 |
| Heparins | 0.60 | 0.17-3.86 | 0.51 | n.a. | n.a. | n.a. |
| AC & AP | 0.81 | 0.40-1.87 | 0.59 | 1.13 | 0.22-5.0 | 0.88 |

AC: Anticoagulation; AP: Antiplatelet; DOAC: Direct oral anticoagulant; ESI: Emergency Severity Index; GCS: Glasgow Coma Scale; hAIS: head specific abbreviated injury scale; INR: international normalized ratio; ISS: injury severity scale; OAC: oral anticoagulant; tICH: traumatic intracranial hemorrhage; n.a.: not applicable.

**Supplementary Table 3.** Patients’ characteristics with tICH and negative protein S100b serum concentrations (n=10).

| **Patient ID** | **gender** | **Age (years)** | **Delay from fall** | **Compartment of ICH** | **hAIS** | **neurosurgery** | **Anticoagulation/**  **Antiplatelet therapy** | **mortality** |
| --- | --- | --- | --- | --- | --- | --- | --- | --- |
| 511 | female | 87 | ≤24 h | SAB | 2 | no | AP single | no |
| 776 | female | 65 | ≤24 h | SAB | 2 | no | no | no |
| 1092 | female | 69 | ≤24 h | SDH & SAB | 5 | yes | OAC | no |
| 1438 | female | 85 | ≤24 h | SDH | 3 | no | no | no |
| 1528 | female | 80 | ≤24 h | SDH & SAB | 3 | no | no | no |
| 1895 | male | 80 | ≤24 h | SDH | 3 | no | AP single | no |
| 1904 | male | 85 | ≤24 h | SDH | 3 | no | AP single | no |
| 2025 | female | 67 | ≤24 h | SAB | 2 | no | no | no |
| 2102 | male | 96 | ≤24 h | IPH | 2 | no | no | no |
| 4171 | male | 65 | ≤24 h | SAB & IPH | 2 | no | no | no |

AP: antiplatelet therapy; hAIS: head specific abbreviated injury scale; IPH: intraparenchymal hemorrhage; OAC: oral anticoagulation; S100b: Protein S100b; SAB: subarachnoidal hemorrhage; SDH: subdural hematoma; tICH: traumatic intracranial hemorrhage.


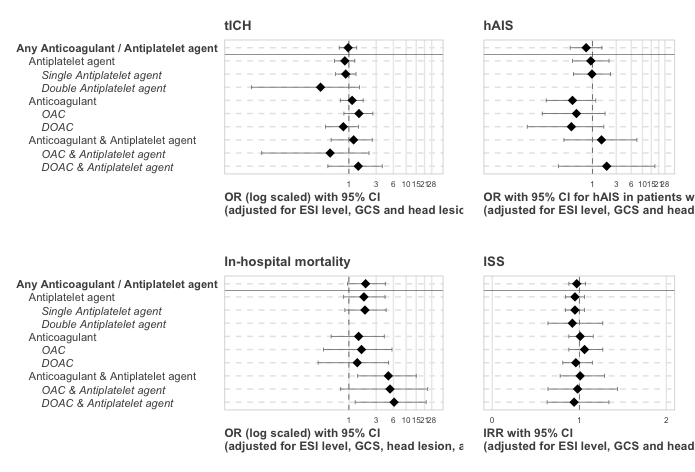
**Supplementary Figure 1.** Multivariable quasi-Poisson regression models adjusted for Emergency Severity Index level, Glasgow Coma Scale, and injury signs above the clavicle, depicting the association between anticoagulation/antiplatelet therapies and ordinal (intracranial hemorrhage, in- hospital mortality) and continuous (head-specific Abbreviated Injury Scale, Injury Severity Score) outcomes as odds ratio (OR), and incidence rate ratios (IRR) with 95% confidence interval (CI), respectively. DOAC, direct oral anticoagulant; OAC, oral anticoagulant.
